# Polystyrene Microplastics Induce Photosynthetic Impairment in *Navicula* sp. at Physiological and Transcriptomic Levels

**DOI:** 10.3390/ijms26010148

**Published:** 2024-12-27

**Authors:** Xi Li, Zunyan Wang, Yiyong Chen, Qi Li

**Affiliations:** 1 College of Urban and Environmental Sciences, Northwest University, Xi’an 710127, China; xili@nwu.edu.cn (X.L.); scsi@nwu.edu.cn (Z.W.); 2 Shaanxi Key Laboratory of Earth Surface System and Environmental Carrying Capacity, Xi’an 710127, China; 3 Research Center for Eco-Environmental Sciences, Chinese Academy of Sciences, Beijing 100085, China

**Keywords:** polystyrene microplastics, *Navicula* sp., molecular mechanism, toxic effects, transcriptomics

## Abstract

The rising concentration of microplastics (MPs) in aquatic environments poses increasing ecological risks, yet their impacts on biological communities remain largely unrevealed. This study investigated how aminopolystyrene microplastics (PS-NH_2_) affect physiology and gene expression using the freshwater alga *Navicula* sp. as the test species. After exposing *Navicula* sp. to high PS-NH_2_ concentrations for 24 h, growth was inhibited, with the most significant effect seen after 48 h. Increasing PS-NH_2_ concentrations reduced chlorophyll content, maximum photochemical quantum yield (Fv/Fm), and the photochemical quenching coefficient (Qp), while the non-photochemical quenching coefficient (NPQ) increased, indicating a substantial impact on photosynthesis. PS-NH_2_ exposure, damaged cell membrane microstructures, activated antioxidant enzymes, and significantly increased malondialdehyde (MDA), glutathione peroxidase (GPX), and superoxide dismutase (SOD) activities. Transcriptomic analysis revealed that PS-NH_2_ also affected the gene expression of *Navicula* sp. The differentially expressed genes (DEGs) are mainly related to porphyrin and chlorophyll metabolism, carbon fixation in photosynthesis, endocytosis, and glycolysis/gluconeogenesis. Protein–protein interaction (PPI) analysis revealed significant interactions among DEGs, particularly within photosystem II. These findings shed insights into the toxic mechanisms and environmental implications of microplastic interactions with phytoplankton, deepening our understanding of the potential adverse effects of microplastics in aquatic ecosystems.

## 1. Introduction

Plastics, with their advantageous properties like low density, thermal and electrical conductivity, and corrosion resistance, have become widespread in various industries. Global plastic production in 2018 reached 359 million tons, with China alone producing 108 million tons [1,2]. However, inadequate recycling and disposal practices have led to the accumulation of plastic waste in the environment, giving rise to a new class of pollutants known as microplastics (MPs) [3,4,5]. Characterized by their small particle size (<5 μm), MPs readily enter organisms upon ingestion or absorption, affecting their morphology and metabolic processes. With a large specific surface area, MPs can adsorb pollutants and serve as carriers for their transmission, accumulating in organisms and disrupting ecosystem structure and function [6]. Nanoplastics (NPs), which are defined as plastic particles smaller than 1 μm, pose an even greater threat for living organisms due to their unique properties. Unlike larger MPs, NPs can penetrate biological barriers such as plant cell walls and membranes. Exposure to MPs and NPs has been associated with negative effects on organism health, and their ability to adsorb and transport organic pollutants further exacerbates environmental contamination, highlighting the urgent need to address the risks posed by MPs to both ecosystems and human health.

Previous research has highlighted the harmful effects of MPs on planktonic algae, zooplankton, and fish [7,8,9]. Among single-celled diatoms, *Navicula* sp. is widely used as a standard test organism in water pollution monitoring and aquatic toxicity studies. Exposure to MPs inhibits the growth and photosynthesis of planktonic algae, leading to the production of reactive oxygen species and oxidative damage [10,11,12]. High concentrations of PVC and PP reduce the FV/Fm and F_v_/F_0_ values in *Chlorella* and *Microcystis floralis*, thereby inhibiting their photosynthetic activity [13,14]. Zhang et al. (2017) examined the effects of powdered (mPVC) and flake (bPVC) polyvinyl chloride, with particle sizes of 1 μm and 1 mm, respectively, on the growth of *Osteophylla* [11]. After 96 h of exposure, mPVC significantly inhibited *Osteophylla*’s growth by 39.7%. High-concentration (50 mg/L) mPVC treatment also reduced chlorophyll content and photosynthetic efficiency. In summary, algae are more sensitive to pressures from MPs compared to higher plants [15]. As primary producers, algae play a crucial role in aquatic ecosystems by converting inorganic substances into organic matter through photosynthesis, thereby providing energy and nutrients for aquatic organisms and contributing to the biogeochemical cycling of nutrients [16]. The stress exerted by microplastics (MPs) on algae could have cascading effects on aquatic ecosystems. Therefore, it is important to explore the impact of MPs on algae.

Although there is increasing concern about how MPs are impacting aquatic ecosystems, this subject is lacking regarding freshwater environments. This knowledge gap is particularly critical given the ecological importance of planktonic algae, which serve as primary producers in aquatic food webs. Although some studies have highlighted the toxic effects of MPs on aquatic algae [15], the underlying mechanisms driving these toxic responses have not been examined in detail [17]. Addressing this gap is essential for understanding the ecological risks associated with microplastic pollution. In view of this, the present study selected *Navicula* sp., a common freshwater planktonic alga, as a model organism to evaluate the effects of MP exposure. We applied NPs-NH_2_ at concentrations of 0–10 mg/L to investigate their effects on *Navicula* sp. These concentrations were chosen to simulate potential environmental contamination levels based on reported ranges in contaminated water bodies [18,19]. We investigated the impact of MPs on the growth and metabolism of *Navicula* sp., focusing on its growth characteristics, photosynthetic parameters, and oxidative stress responses. Additionally, RNA sequencing (RNA-seq) was employed to identify genes and intracellular pathways involved in the algal stress response induced by MPs [20,21]. These findings provide valuable insights into the physiological mechanisms underlying algal responses to MP exposure and hold significant implications for advancing our understanding of microplastics-algae interactions, conducting ecological risk assessments, and improving environmental monitoring and pollution management strategies.

## 2. Results

### 2.1. Acute Toxic Effects of PS-NH_2_ on Navicula sp.

**Figure 1:**
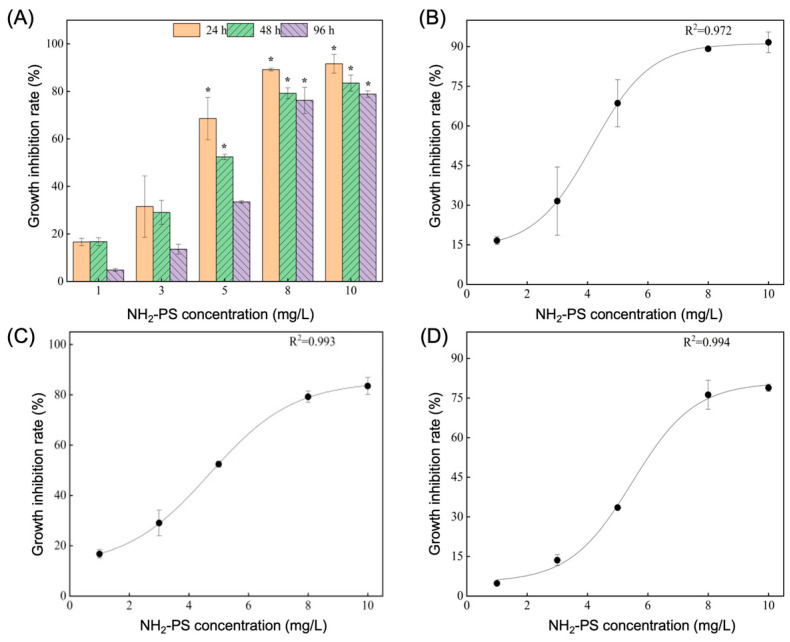
Effects of varying PS-NH_2_ concentrations on *Navicula* sp. growth inhibition rate: (**A**) growth inhibition rate; (**B**–**D**) dose–response curves at 24 h, 48 h, and 96 h, respectively. “*” denotes statistical significance (*p* < 0.05) between the experimental and control groups, and data are expressed as mean ± standard error (*n* = 3).

Table S2 displays the growth status of *Navicula* sp. after exposure to various durations of PS-NH_2_. The EC_50_ values for PS-NH_2_ on *Navicula* sp. at 24, 48, and 96 h were 4.16, 4.67, and 5.48 mg/L, respectively. Figure 1 illustrates the growth inhibition rates of *Navicula* sp. under different PS-NH_2_ concentrations and exposure durations, indicating a substantial inhibitory effect of PS-NH_2_ on *Navicula* sp. growth. The inhibition rate increased steadily with higher PS-NH_2_ concentrations, reaching 90% as the concentration rose from 1 to 8 mg/L. However, there was no significant additional increase when the concentration increased from 8 to 10 mg/L. Disparities in growth inhibition rates were observed among exposure groups when PS-NH_2_ concentrations exceeded 5 mg/L, with a gradual decline in inhibition rates with extended exposure (Figure 1A). PS-NH_2_ showed a pronounced inhibitory effect on *Navicula* sp. at higher concentrations (8–10 mg/L), but less so at lower concentrations (below 3 mg/L, Figure 1B–D).

### 2.2. Analysis of Photosynthetic Characteristics

**Figure 2:**
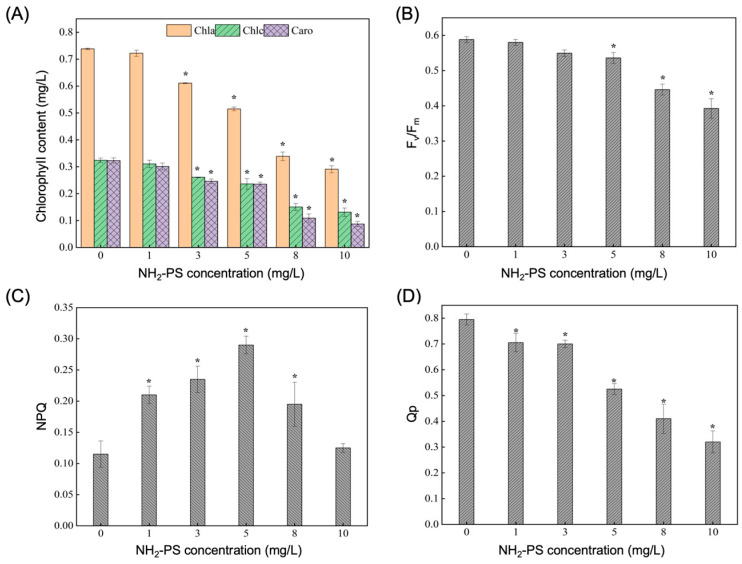
Impact of different PS-NH_2_ concentrations on *Navicula* sp. photosynthetic parameters: (**A**) chlorophyll content; (**B**) Fv/Fm; (**C**) NPQ; (**D**) Qp. “*” indicates a significant difference (*p* < 0.05) between the experimental and control groups, and data are expressed as mean ± standard error (*n* = 3).

Figure 2A depicts the impact of PS-NH_2_ on photosynthetic parameters in *Naviculata* sp. Exposure to 1 mg/L of PS-NH_2_ did not significantly affect chlorophyll content in *Naviculata* sp. However, exposure to 3, 5, 8, and 10 mg/L of PS-NH_2_ resulted in substantial decreases in chlorophyll-a content by 17.24%, 30.25%, 54.11%, and 60.61%, respectively (*p* < 0.05). Chlorophyll-c content also exhibited significant reductions of 19.51%, 27.15%, 53.52%, and 59.57% (*p* < 0.05), while carotenoid content displayed significant decreases of 23.87%, 27.18%, 66.20%, and 73.10%, respectively (*p* < 0.05, Figure 2A). At lower concentrations of PS-NH_2_ (1 and 3 mg/L), there was no significant effect on the maximum photochemical quantum yield (Fv/Fm) of *Naviculata* sp. However, following exposure to 5, 8, and 10 mg/L PS-NH_2_, the Fv/Fm significantly decreased by 8.84%, 24.15%, and 33.25%, respectively (*p* < 0.05, Figure 2B), indicating increasing inhibition of Fv/Fm in *Naviculata* sp. with rising PS-NH_2_ concentration. Non-photochemical quenching (NPQ), a metric estimating the dissipation of light energy through thermal pathways in phytoplankton, was significantly affected by PS-NH_2_ in *Naviculata* sp. NPQ increased with the rising PS-NH_2_ concentration, from 1 to 8 mg/L, followed by a subsequent decline (*p* < 0.05, Figure 2C). At an exposure concentration of 5 mg/L, NPQ peaked at approximately 0.29, a 2.52-fold increase compared to the control group. Interestingly, NPQ did not exhibit significant changes under the 10 mg/L PS-NH_2_ treatment. The photochemical quenching coefficient (Qp), reflecting the photosynthetic activity of algal cells, gradually and significantly reduced with the increasing PS-NH_2_ concentration (*p* < 0.05, Figure 2D), reaching a minimum value of 0.32 at an exposure concentration of 10 mg/L, representing a 59.75% reduction.

### 2.3. Activity of Antioxidant Enzymes in Navicula sp.

**Figure 3:**
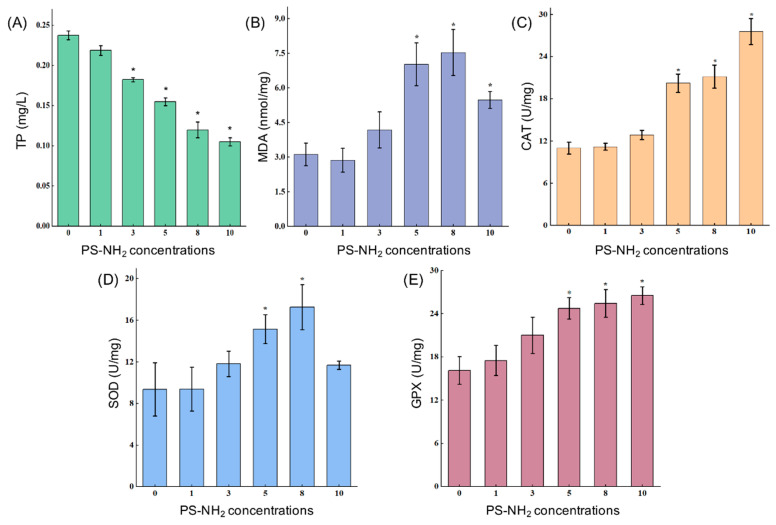
Influence of varying PS-NH_2_ concentrations on antioxidative enzymes in *Navicula* sp. (**A**) TP; (**B**) MDA; (**C**) CAT; (**D**) SOD; (**E**) GPX. “*” denotes significant differences (*p* < 0.05) between the experimental and control groups, and data are expressed as mean ± standard error (*n* = 3).

Figure 3 depicts the impact of PS-NH_2_ on the antioxidative enzyme system in *Navicula* sp. The total protein concentration in *Navicula* sp. decreased with the increasing PS-NH_2_ concentration. Specifically, at PS-NH_2_ concentrations of 3–10 mg/L, the total protein concentration was significantly lower compared to the control group (*p* < 0.05). Remarkably, concentrations of 8 mg/L and 10 mg/L led to a significant reduction in the total protein concentration in *Navicula* sp. by 49.51% and 55.82%, respectively (Figure 3A). CAT and GPX levels showed a substantial increase, with an increasing PS-NH_2_ concentration (Figure 3C,E). In the presence of PS-NH_2_, at concentrations ranging from 5 to 10 mg/L, CAT activity was 1.84-fold, 1.92-fold, and 2.50-fold higher than that of the control group, while GPX activity increased by 1.53-fold, 1.58-fold, and 1.64-fold compared to the control group. Similarly, MDA and SOD enzyme activities in PS-NH_2_-treated *Navicula* sp. demonstrated an increasing trend (*p* < 0.05, Figure 3B,D). Specifically, at PS-NH_2_ concentrations of 5–8 mg/L, MDA levels in *Navicula* sp. were 2.26-fold, 2.42-fold, and 1.76-fold higher than that in the control group, respectively. The SOD levels increased by 1.62-fold, 1.84-fold, and 1.25-fold compared to the control group. However, at 10 mg/L PS-NH_2_, both MDA and SOD activities in *Navicula* sp. showed a declining trend.

### 2.4. Transcriptome Sequencing, Assembly and Analysis of DEGs

A comparative transcriptome analysis was conducted on nine *Navicula* sp. samples (three from each of the control, low-concentration, and high-concentration groups) to assess the molecular impact of PS-NH_2_ exposure. The analysis yielded 3.804 × 10^8^ raw reads, which were pruned and assessed for quality, resulting in 131, 113, 370, 126, 882, 620, and 116, 184, and 246 clean reads from the control, high-concentration, and low-concentration groups, respectively. Sequencing quality was high, with over 97.50% of bases meeting the Q20 quality threshold, and over 93.50% meeting the Q30 threshold for each sample. The average GC content was approximately 47.90%.

**Figure 4:**
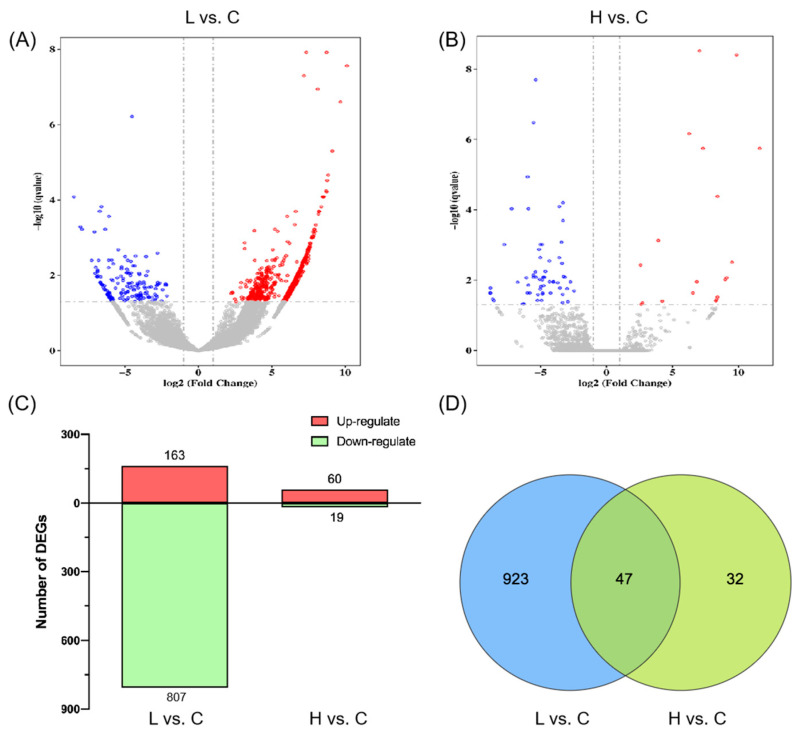
(**A**,**B**) Volcano plot illustrating DEGs between control and exposure groups. The *x*-axis displays log_2_FC (fold-change), and the *y*-axis displays −log_10_ (*q*-value). Red represents significantly upregulated genes, blue represents significantly downregulated genes, gray represents insignificantly expressed genes, with each circle representing one gene. (**C**) Number of DEGs; (**D**) Venn diagram depicting common and unique DEGs in response to the two stressors. C: Control group; L: low concentration; H: high concentration.

Correlation testing, depicted in Figure S3A, showed a correlation coefficient near 0.80 for repeated samples in all groups. Biological replicates in each group were closely correlated but notably distinct from those in other groups, indicating the reproducibility and reliability of the RNA-seq data. Figure S3B illustrates the expression pattern of DEGs in all groups. In the low-concentration group, 970 DEGs were identified, with 163 up-regulated and 807 down-regulated. The high-concentration group had 79 differential genes, including 60 up-regulated and 19 down-regulated genes (Figure 4A–C). A Venn diagram (Figure 4D) showed 47 DEPs commonly shared between the two treatments.

### 2.5. GO and KEGG Pathway Enrichment Analysis

**Figure 5:**
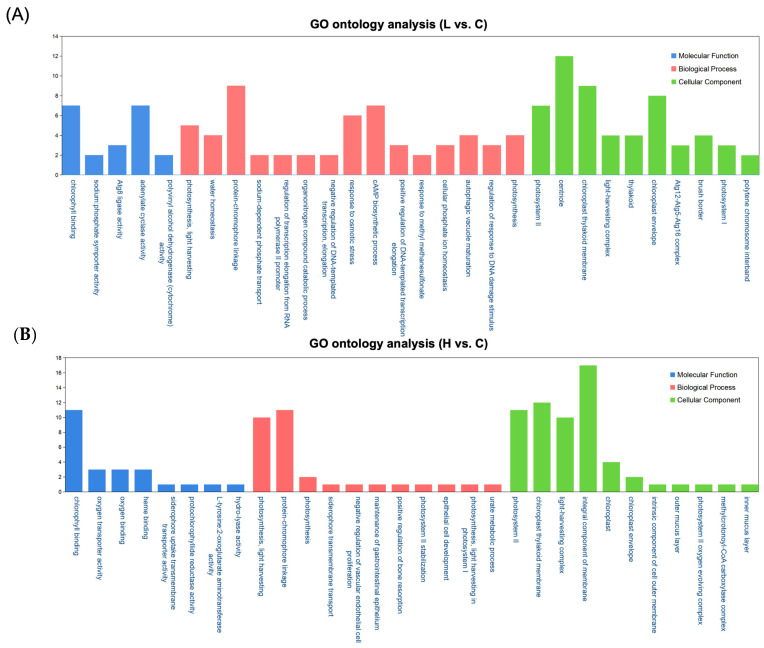
Histogram displaying enriched subcategories from GO analysis of DEGs in *Navicula* sp. after exposure to (**A**) Low concentration and (**B**) High concentration of PS-NH_2_. The *x*-axis presents GO terms related to the main ontologies (biological process, cellular component, and molecular function), while the *y*-axis indicates the number of genes. C: Control group; L: low concentration; H: high concentration.

The DEGs were categorized into GO terms across biological processes, cellular components, and molecular functions, as shown in Figure 5. In the low-concentration group, DEGs were significantly enriched in categories such as chlorophyll binding, adenylate cyclase activity, protein-chromophore linkage, photosystem II, and the chloroplast thylakoid membrane (Figure 5A). Similarly, the high-concentration group showed significant GO term enrichments, focusing on chlorophyll binding, protein–chromophore linkage, photosynthesis, light harvesting, and integral components of the membrane (Figure 5B). These results underscore the key functions of DEGs in both low and high-concentration groups, highlighting their roles in photosynthesis, chloroplast function, and protein synthesis and transport.

Furthermore, the KEGG enrichment analysis of the DEGs revealed their potential involvement in various pathways, as presented in Supplemental Table S3. There is notable emphasis on processes related to porphyrin and chlorophyll metabolism, carbon fixation in photosynthesis, endocytosis, glycolysis/gluconeogenesis, and endocytosis pathways (Figure S4).

### 2.6. Protein–Protein Interaction Analysis

**Figure 6:**
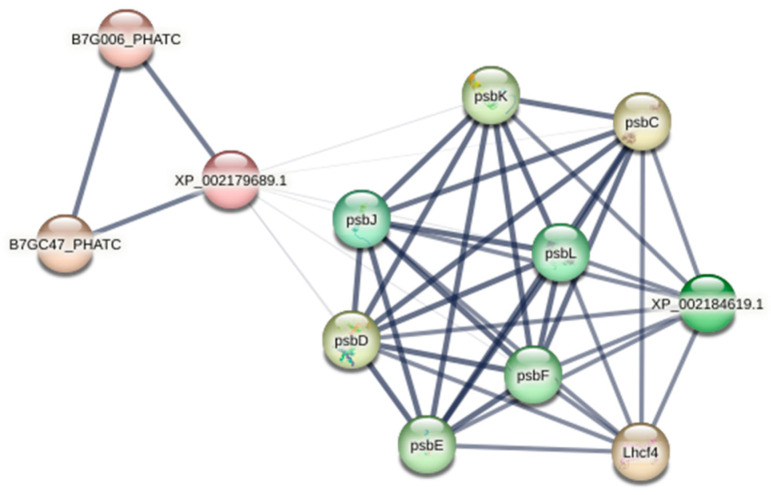
Analysis of protein–protein interactions among differentially abundant genes in *Navicula* sp. under PS-NH_2_ exposure stress. Each node corresponds to the protein encoded by the respective gene, and line thickness indicates data support strength.

The network program, STRING, was employed to analyze protein–protein interactions among 47 DEGs that significantly varied under both concentration stresses (Figure 6). After removing unconnected nodes, an interaction network emerged, highlighting chloroplast genes psbL and psbJ, as well as the pivotal gene psbD linked to Photosystem II. These gene interconnections play a crucial role in regulating plant photosynthesis.

## 3. Discussion

The ecological impact of nanoplastics has raised global concerns, prompting investigations into their biological effects [22,23]. Previous studies have demonstrated that nanoplastics can affect phytoplankton growth, photosynthesis, oxidative stress, and gene expression, with their impact influenced by nanoplastic size, exposure concentration, and duration [10,11]. However, the molecular mechanisms underlying nanoplastic-induced growth inhibition in *Navicula* sp. remain unclear. Transcriptomics provides a valuable tool for predicting pollutant toxicity and understanding microplastic effects [24,25]. Therefore, we employed a comparative transcriptomics approach to elucidate the potential molecular mechanisms in *Navicula* sp. under PS-NH_2_ stress at both physiological and transcriptomic levels.

Our study demonstrates that increasing PS-NH_2_ concentration leads to higher growth inhibition rates in *Navicula* sp., with growth nearly halted at concentrations of 8–10 mg/L. This suggests that significant growth inhibition primarily occurs at high concentrations, which is consistent with previous findings. Wang et al. (2021) found no significant microalgae growth inhibition at microplastic concentrations of up to 2500 mg/L [26], while Besseling et al. (2014) observed algal growth inhibition by nanosized PS microplastics only at concentrations exceeding 44 mg/L [27]. This highlights the concentration-dependent nature of microplastic-induced algae inhibition. Even at a low PS-NH_2_ concentration of 5 mg/L, our study showed significant growth inhibition, indicating *Navicula* sp.’s sensitivity to PS-NH_2_ toxicity. Furthermore, prolonged exposure led to a gradual decline in the growth inhibition rate, suggesting that *Navicula* sp. may have developed mechanisms to mitigate PS-NH_2_-induced stress. Research confirms that algal cells produce extracellular polymers when exposed to stress, which can absorb microplastics, thereby resisting microplastic-induced stress [28].

Photosynthetic characteristics are vital indicators of changes in algal physiology due to nanoplastic exposure [29]. Chlorophyll content, a key indicator of photosynthesis [30], decreased significantly with higher PS-NH_2_ exposure in *Navicula* sp., indicating disrupted photosynthesis at high concentrations. This aligns with prior studies showing pronounced chlorophyll impacts at high microplastic concentrations, contrasting with minimal effects at lower levels [31]. High concentrations likely disrupt chloroplast ultrastructure, affecting photosynthesis [12]. Under low PS-NH_2_ stress (<5 mg/L), the chlorophyll fluorescence parameter Fv/Fm remained stable. However, above 5 mg/L, Fv/Fm decreased significantly, indicating stress and PSII instability in *Navicula* sp. in accordance with what has been observed in *Chlorella* [32]. NPQ increased significantly from 1 to 8 mg/L of PS-NH_2_ but remained unchanged at 10 mg/L, suggesting that *Navicula* sp. activates photoprotective mechanisms up to a threshold, potentially losing this response at higher stress levels, indicating that adaptive photosynthetic mechanisms cope with PS-NH_2_ stress [12]. Furthermore, higher PS-NH_2_ concentrations reduced electron transfer activity in PSII, likely due to physical disruption of *Navicula* sp. cell surfaces by microplastics. PS-NH_2_ is believed to penetrate cell membranes, damaging cell and organelle morphology, reducing electron transfer efficiency and photosynthetic energy use [33,34]. PS-NH_2_ adhesion to *Navicula* sp. cells reduces material and energy exchange, negatively impacting respiration and photosynthesis [35]. *Navicula* sp., with a typical pore size of around 50 nm, can internalize microplastics [36], suggesting PS-NH_2_ could significantly disrupt algal growth by affecting photosynthetic capacity once internalized [28].

Excessive levels of reactive oxygen species (ROS) induced by nanoplastics can disrupt an organism’s internal equilibrium, hindering protein anabolism and affecting protein structure and composition [37]. This study examines the impact of PS-NH_2_ stress on *Navicula* sp., revealing disruptions in intracellular metabolism and protein synthesis inhibition. Previous studies have demonstrated that nanoplastics can trigger ROS production and accumulation in algal cells, leading to cell lipid peroxidation [38]. MDA serves as a reliable biomarker of lipid peroxidation in algal cells, indicating their oxidative stress levels [39]. Under low PS-NH_2_ stress, MDA content remained stable, suggesting that *Navicula* sp. activated an antioxidant protection mechanism [10]. However, under high PS-NH_2_ stress, MDA content significantly increased, indicating that there was a disruption of its antioxidant enzyme system [40]. The antioxidant enzyme system plays a crucial role in protecting plant macromolecules from ROS-induced damage [41]. Previous studies have shown that PSNPs can enhance plant antioxidant enzyme activity, aligning with our findings [42,43]. Exposure to PS-NH_2_ concentrations above 5 mg/L significantly increased maize SOD, CAT, and GPX activities, suggesting that stressed *Navicula* sp. mitigated membrane lipid peroxidation by boosting intracellular antioxidant enzyme activity. Wan et al. (2015) observed no significant effects on SOD and CAT activities in microcystin-producing algae at low erythromycin concentrations, indicating that oxidative stress in microalgal cells occurs only when stress exceeds the specific threshold required to maintain oxidant–antioxidant enzyme balance [44]. Specifically, when the PS-NH_2_ exposure concentration reached 10 mg/L, SOD activity did not significantly increase, suggesting excessive PS-NH_2_ disrupted specific SOD synthesis pathways [13].

Transcriptomics is essential for understanding plant stress responses’ gene regulatory mechanisms. Transcriptome sequencing of PS-NH_2_-exposed *Navicula* sp. revealed that most DEGs were linked to porphyrin and chlorophyll metabolism pathways in diatoms (Figure S5). Genes involved in chlorophyll synthesis and metabolism (Por, chlP, and bchP) were significantly downregulated, indicating reduced chlorophyll synthesis and metabolism. Previous studies suggest that chlorophyll possesses antioxidant properties; downregulation of its synthesis genes can lead to ROS accumulation, hindering diatom growth [45]. Low-concentration stress upregulated genes related to CO_2_ fixation (E4.1.1.49, pckA, GAPDH, gapA), suggesting that *Navicula* sp. enhances carbon fixation in photosynthesis to sustain growth under stress (Figure S6). Similarly, genes associated with glycolysis/gluconeogenesis (GAPDH, pckA, and ACSS1_2) were upregulated, providing more energy for antioxidant enzyme synthesis (Figure S7). Previous studies have indicated that glycolysis-related genes can help algae mitigate oxidative stress and alleviate cell membrane damage, helping alleviate oxidative stress and cell membrane damage [46,47,48,49,50]. It can be hypothesized that this upregulation also provides energy for *Navicula* sp. to respond to external stimuli [48]. PS-NH_2_ stress significantly altered the endocytosis pathway (Figure S8), a major interaction route for microalgal cells with nanomaterials [51,52,53]. Our results confirm that endocytosis accelerates PS-NH_2_ accumulation on chloroplasts and the nucleus, leading to algal cell apoptosis [54]. Additionally, network analysis of DEGs responding to both stress concentrations shows PS-NH_2_ interferes with *Navicula* sp.’s photosynthetic photosystem II activity, reducing intracellular electron transfer and photosynthesis energy (Figure 6). This study reveals that PS-NH_2_ disrupts photosynthesis in *Navicula* sp.

## 4. Materials and Methods

### 4.1. Polystyrene Microplastics Pretreatment and Characterization

PS-NH_2_, sourced from Baioutai Biotechnology Co., Ltd., (Beijing, China), comprised monodisperse polystyrene microspheres with a 50 nm diameter, and were supplied at a concentration of 2.5% (*w*/*v*). The size distribution of PS-NH_2_ was determined using a Mastersizer Laser Particle Sizer (MLPS, Mastersizer 3000, Malvern Panalytical Ltd., Malvern, UK). To prevent PS-NH_2_ aggregation, a numerically-controlled ultrasonic cleaner (GD420HTD, Shenzhen Guangdian Ultrasonic Equipment Co., Ltd., Shenzhen, China) was utilized at a frequency of 40 kHz for 10 min. The morphology of PS-NH_2_ was analyzed using high-resolution field emission Transmission Electron Microscopy (TEM, FEI Talos F200X, Thermo Fisher Scientific, Waltham, MA, USA). The characterization results of PS-NH_2_ suspensions are presented in Figure S1.

### 4.2. Navicula sp. Culture and Exposure to PS-NH_2_

*Navicula* sp. (FACHB-1996) was sourced from the Freshwater Algae Culture Collection at the Institute of Hydrobiology (National Aquatic Biological Resource Center, Wuhan, China) and cultured in a CSI medium, as per the specified requirements (Table S1). The pH of the CSI medium was adjusted to 7.0 ± 0.2 using 1 mol/L dilute hydrochloric acid before autoclaving at 121 °C for 30 min in a vertical autoclave. It was then securely sealed and covered with gauze and cotton plugs. Aseptic conditions were maintained by disinfecting the operating table with 30 min of ultraviolet radiation. Algae were cultivated in a temperature-controlled incubator at 25 ± 0.5 °C, with a light intensity of 2500 Lux and a 12 h/12 h light–dark cycle in 250 mL Erlenmeyer flasks, gently agitated 6–8 times daily for optimal growth.

*Navicula* sp., in its logarithmic growth phase, was aseptically introduced into 250 mL Erlenmeyer flasks, each containing 100 mL of a culture medium. The initial algae density was 1.0 × 10^4^ cells/mL, with three parallel groups cultured accordingly. On the fifth day, specified concentrations of PS-NH_2_ (0, 1, 3, 5, 8, and 10 mg/L) were added directly to the Erlenmeyer flasks, along with a solvent control group to assess potential microplastic interference [55].

Algal cell counts were determined using a spectrophotometer and platelet count (Figure S1). A linear regression equation was derived from the number of cells and absorbance (OD_680_) to calculate cell density (Figure S2) [56]:

(1) $$Y=1.6976{\;\times \;10}^{7}X\;-\;2.5969{\;\times \;10}^{4}{,\;R}^{2}=0.9998$$

Here, Y represents the density of algae cells (cells/mL), and X denotes the value of algae OD_680_ after microplastic OD_680_ removal.

A 2.5 mL mixture was homogenized to establish the baseline absorbance for the CSI medium. The solution’s absorbance was measured using a UV-Vis spectrophotometer at 680 nm. The regression equation was applied to compute the corresponding cell density, enabling the construction of a growth curve. Following a 48 h exposure of *Navicula* sp. to microplastics, the inhibition rate of algal growth was calculated:

(2) $$\mu_{i-j}=\frac{{\mathrm{lnY}}_{j}-{\mathrm{lnY}}_{i}}{t_{j}-t_{i}}$$

(3) $$I_{r}=\frac{\mu_{C}-\mu_{T}}{\mu_{C}}\times 100$$

In Equation (2), µ_i−j_ represents the specific growth rate from day i to j, with Y_i_ and Y_j_ as the concentrations of algal cells (cells/mL) at times i and j, respectively. Equation (3) defines I_r_ as the growth inhibition rate based on the specific growth rate, where μ_C_ is the average growth rate of parallel samples in the control group, and μ_T_ is the average growth rate of parallel samples in the experimental group.

### 4.3. Quantification of Photosynthetic Parameters

Chlorophyll (Chl) content was determined following Dai’s method (2013) [57]. Algal liquid (10 mL) was filtered through a 0.45 μm fiber filter and transferred to a 50 mL centrifuge tube. Then, 10 mL of acetone solution was added, and the tube was refrigerated at 4 °C for 24 h. After centrifugation at 3000 rpm for 10 min, the supernatant was filtered through a needle-type water filtration membrane. The absorbance at OD_665_, OD_650_, and OD_470_ was measured using a UV-visible spectrophotometer to calculate Chlorophyll a (Chla, mg/L), Chlorophyll c (Chlc, mg/L), and Carotenoid (Caro, mg/L) contents:

(4) $$\mathrm{Chla}\;=\;11.47{\mathrm{OD}}_{665}-0.4{\mathrm{OD}}_{650}$$

(5) $$\mathrm{Chlc}\;=\;24.36{\mathrm{OD}}_{650}-3.73{\mathrm{OD}}_{665}$$

(6) Caro = 1000OD470−2.05Chla221

Following 48 h of exposure to microplastics, 10 mL of algal solution was shielded from light using tin foil in a 50 mL centrifuge tube. Each measurement was preceded by a 15 min dark treatment. Photosynthetic parameters were assessed using a hand-held chlorophyll fluorometer (AquaPen, Photon Systems Instrument, Drásov, Czech Republic). FV/Fm represents the maximum photochemical efficiency of photosystem II (PSII), indicating the energy capture efficiency of the PSII reaction center [58]. The non-photochemical quenching coefficient (NPQ) signifies the ratio of energy used for heat dissipation in PSII to the total energy absorbed by PSII, while the photochemical quenching coefficient (Qp) represents the ratio of energy involved in the photochemical reaction in PSII to the total energy absorbed by PSII [7,59].

### 4.4. Activity Assessment of Antioxidative Enzymes

Following 48 h of PS-NH_2_-induced stress, a 10 mL algal fluid sample was subjected to low-temperature ultracentrifugation (TGL-16, Changsha Yingtai Instrument Co., Ltd., Changsha, China) at 10,000 rpm for 5 min, and the resulting supernatant was discarded. The sediment of *Navicula* sp. was mixed with 2 mL of 0.9% normal saline, centrifuged at 1000 rpm for 5 min, and the supernatant was carefully aspirated with a needle tube, repeating this process thrice. After rinsing, 1 mL of 0.9% saline was added to each sample, mixed thoroughly, and transferred to a 5 mL centrifuge tube. The mixture underwent fragmentation using an Ultrasonic cell crusher (JY92-IIN, Ningbo Xinyi Ultrasonic Equipment Co., Ltd., Ningbo, China) for 4 min, with a working time of 2 s and an interval of 3 s. The centrifuge tubes were then placed in an ice-water bath to inhibit enzymatic decomposition, and the resulting slurry was used for the subsequent determination of oxidative stress markers.

Total Protein (TP), Malondialdehyde (MDA), Catalase (CAT), Superoxide Dismutase (SOD), and Glutathione Peroxidase (GPX) were quantified using commercially procured biomarker assay kits (Nanjing Jiancheng Bioengineering Institute, Nanjing, China). The algal homogenate supernatant, prepared in advance, was utilized for MDA (Thiobarbituric acid method), CAT (Visible spectrophotometer method), SOD (Water-soluble tetrazolium method, WST-1), GPX (Colorimetric method), and TP (Visible spectrophotometer method) analysis [60]. Enzyme activity was assessed using a multi-function microplate reader (Tecan Infinite^®^ 200 Pro, Tecan Austria GmbH, Grödig, Austria) at wavelengths of 532 nm, 405 nm, 412 nm, 450 nm, and 562 nm, respectively.

### 4.5. Total RNA Extraction and Sequencing

The culture conditions for *Navicula* sp. remained consistent with those described in Section 2.2. A concentration gradient of 0, 3, and 5 mg/L was established for the transcriptome experiment based on the EC50 values. Six biological replicates were prepared for each concentration gradient. After 48 h of incubation under these conditions, the algal solution underwent centrifugation at 10,000 rpm for 10 min, and the supernatant was discarded. The algal liquid samples for the three concentrations were labeled as C (Control: 0 mg/L), L (low concentration: 3 mg/L), and H (high concentration: 5 mg/L). All samples were snap-frozen using liquid nitrogen and stored at −80 °C.

Total RNA extraction from each *Navicula* sp. sample was performed using the TRIzol Reagent kit. Quantification and identification of total RNA from each sample were carried out using an Agilent 2100 Bioanalyzer (Agilent Technologies, Palo Alto, CA, USA) and a NanoDrop (Thermo Fisher Scientific Inc.). The samples exhibited a relatively complete RNA extraction process. An RNA-seq library was constructed and sequenced by GENEWIZ Biotechnology Company, utilizing the Novaseq platform (Illumina Inc., San Diego, CA, USA).

### 4.6. Transcriptome Assembly and Analysis of Differentially Expressed Genes

Raw reads underwent a transformation into FASTQ format using Illumina’s offline base caller (v1.6). Trinity version v2.0.6 was utilized to assemble a de novo transcriptome of *Navicula* sp., with longer contigs assembled until further extension was not possible. Removal of redundant transcripts yielded individual genes. The final output, comprising predicted transcript files, was employed for subsequent analyses. Assembled transcripts were compared against several National Center for Biotechnology Information (NCBI) protein databases, including NR, NT, PFAM, GO, and Swiss-Prot, using BLASTx (E-value threshold = 1 × 10^−5^). Differentially expressed genes (DEGs) between the treated and control groups were analyzed using the DESeq2 package (version 1.26.0) (fold change > 2; adjusted *p*-value < 0.05) [61].

### 4.7. Bioinformatic Analysis

The Gene Ontology (GO) enrichment analysis was conducted using resources from the GO Consortium (http://www.geneontology.org/; accessed on 20 October 2023). Differential gene pathways were annotated and categorized using the Kyoto Encyclopedia of Genes and Genomes (KEGG) pathway database. The Venn diagram online tool was employed to analyze the intersection of DEGs across distinct exposure groups. PPI network analysis of DEGs under low and high PS-NH_2_ exposure conditions was conducted using the publicly accessible program STRING (http://string-db.org/; accessed on 20 October 2023) and Cytoscape software (version 3.7.2) with default parameters [62].

### 4.8. Statistical Analysis

Statistical data were processed using SPSS 26.0 software, and differences among treatment groups were assessed with the One-way ANOVA Dunnett test. Significance, denoted by “*”, was observed between the experimental and control groups (*p* < 0.05). The data are presented as the mean standard error (*n* = 3), and graphs were created using Origin 2021 Pro software.

## 5. Conclusions

This study explores the impact of PS-NH_2_ on freshwater microalgae, specifically *Navicula* sp., focusing on identifying intracellular pathways using RNA-seq analysis. The results reveal a significant, dose-dependent inhibition of *Navicula* sp. growth by PS-NH_2_, with the most substantial growth inhibition observed after 48 h. Additionally, PS-NH_2_ affects the photosynthetic apparatus, resulting in reduced photosynthetic energy, compromised light absorption, and diminished light-harvesting capabilities. An adaptive regulatory feedback mechanism was observed in algae under PS-NH_2_ stress, enhancing electron transfer efficiency in PSII reaction centers to maintain energy allocation equilibrium. Furthermore, all PS-NH_2_ treatment groups exhibit increased activities of CAT, SOD, and GPX, aiming to counterbalance excess ROS. The influence of PS-NH_2_ was not limited to the physiological aspects, it significantly impacted the gene expression of *Navicula* sp. Transcriptomic analysis revealed a total of 1002 differentially expressed genes (DEGs). Specifically, exposure to 3 and 5 mg/L of PS-NH_2_ resulted in 970 DEGs (upregulated: 163, downregulated: 807) and 79 DEGs (upregulated: 60, downregulated: 19), respectively. The transcriptomic results indicate that PS-NH_2_ disrupts photosynthesis-related pathways, including chlorophyll metabolism, carbon fixation, and glycolysis/gluconeogenesis. This disruption involves the upregulation of genes associated with endocytosis, facilitating PS-NH_2_ assimilation by algal cells. Consequently, it can be hypothesized that these cells secrete harmful metabolites, which may trigger algal cell apoptosis. Overall, this study sheds light on the intricate mechanisms of PS-NH_2_’s impact on microalgae, providing novel insights into the ecological risks of nanomicroplastics to aquatic organisms.

## Data Availability

Data is contained within the article and Supplementary Materials.

## Supplementary Materials

The supporting information can be downloaded at: https://www.mdpi.com/article/10.3390/ijms26010148/s1.
