# Supplementary material for: Polystyrene Microplastics Induce Photosynthetic Impairment in Navicula sp. at Physiological and Transcriptomic Levels
Source: Int J Mol Sci. 2024 Dec 27;26(1):148. doi: 10.3390/ijms26010148 (PMC11720487; doi:10.3390/ijms26010148)
Supplement: Supplementary file 1 [file ijms-26-00148-s001.zip › ijms-3373814-supplementary.pdf]

Supplementary Data

Polystyrene microplastics induce photosynthetic impairment in *Navicula sp.* at physiological and transcriptomic levels

Xi Li [a,b], Zunyan Wang [a,b], Yiyong Chen [c*], Qi Li [a,b,*]

[a] *College of Urban and Environmental Sciences, Northwest University, Xi'an, 710127，China.*

[b] *Shaanxi Key Laboratory of Earth Surface System and Environmental Carrying Capacity, Xi'an, 710127，China.*

[C] *Research Center for Eco-Environmental Sciences, Chinese Academy of Sciences, Beijing, China.*

Submitted to *International Journal of Molecular Sciences*

Table S1. EC_{50} of *Navicular sp.* under PS-NH_2 stress
Table S2. Components of CSI medium
Table S3. KEGG pathways significantly enriched (p < 0.05) in *Navicula sp.* exposed to PS-NH_2
Fig. S1. Characterisations of NH_2-PS and a TEM imaging of PS-NH_2.
Fig. S2. Standard curve of *Navicula sp.*
Fig. S3. (A). Characterization of the transcriptome dataset. Principal component analysis (PCA) exhibits clear distinctions between Control and expression group. (B). Volcano map of DEGs in *Navicular sp.* (q-value ⩽ 0.05). C: Control group; H: High concentration group; L: Low concentration group.
Fig. S4. KEGG pathway changes of *Navicular sp.* induced by PS-NH_2. C: Control group; H: High concentration group; L: Low concentration group.
Fig. S5. Transcriptomic analysis of porphyrin and chlorophyll metabolic pathways in *Navicula sp.* after exposure to PS-NH_2.
Fig. S6. Transcriptomic analysis of carbon fixation pathways in photosynthetic organisms after exposure of *Navicula sp.* to PS-NH_2.
Fig. S7. Transcriptomic analysis of the Glycolytic/Glyco-isomeric pathway after exposure to PS-NH_2 in *Navicula sp.*
Fig. S8. Transcriptomic analysis of Endocytosis pathway in *Navicula sp.* exposed to PS-NH_2.

Supplementary tables

Table S1. Components of CSI medium

ID	Component	Dosage	Liquors concentration
1	$Ca(NO_3)_2 \cdot 4H_2O$	1 mL/L	15 g/100 mL dH_2O
2	KNO_3	1 mL/L	10 g/100 mL dH_2O
3	$MgSO_4 \cdot 7H_2O$）	1 mL/L	4 g/100 mL dH_2O
4	β-Na_2glycerophosphate·$5H_2O$	1 mL/L	2.5 g/100 mL dH_2O
5	Vitamin B_{12}	0.1 ug/L	
6	Biotin	0.1 ugL	
7	Thiamine HCl	10 ug/L	
8	HEPES	0.5 g/L	
9	$Na_2SiO_3 \cdot 9H_2O$	0.1 g/L	
10	Soil Extract	30 ml/L	
11（PIV）	Na_2EDTA	6 mL/L	0.75 g/L dH_2O
	$MnCl_2 \cdot 4H_2O$		0.041 g/L dH_2O
	$ZnCl_2 \cdot 7H2O$		0.005 g/L dH_2O
	$Na_2MoO_4 \cdot 2H_2O$		0.004 g/L dH_2O
	$FeCl_3 \cdot 6H_2O$		0.097 g/L dH_2O
	$CoCl_2.6H_2O$		0.002 g/L dH_2O

Note 1: Soil extract: the ratio of soil and distilled water is 200:1 (g/L), sealed with a breathable plug, boiled in a water bath for 3 h, cooled and precipitated for 24 h, repeated three times, filtered to retain the supernatant, sterilized and stored in a refrigerator at 4 ℃ for later use.

Table S2. EC_{50} of *Navicular sp.* under PS-NH_2 stress

Exposed Time	Growth inhibition rates-EC_{50} (mg/L)	R^2
24 h	4.158	0.972
48 h	4.691	0.993
96 h	5.479	0.994

Table S3. KEGG pathways significantly enriched ($p<0.05$) in *Navicula sp.* exposed to PS-NH_2.

Treatment	Pathways	Pathway category	Up-Genes	Down-Genes
Low concentration vs. Control	Ribosome biogenesis in eukaryotes	Translation	*SSUrRNA*	—
	Porphyrin and chlorophyll metabolism	Metabolism of cofactors and vitamins	—	*Por; chlP, bchP*
	Other glycan degradation	Glycan biosynthesis and metabolism	*E3.2.1.25, MANBA, manB*	—
	Endocytosis	Transport and catabolism	*VPS4; HSPA1s*	—
	Phenylpropanoid biosynthesis	Biosynthesis of other secondary metabolites	*katG*	—
	Glycolysis/Gluconeogenesis	Carbohydrate metabolism	*GAPDH, gapA; E4.1.1.49, pckA; ACSS1_2, acs*	—
	Carbon fixation in photosynthetic organisms	Energy metabolism	*GAPDH, gapA; E4.1.1.49, pckA*	—
	Homologous recombination	Replication and repair	*RAD51*	—
	Terpenoid backbone biosynthesis	Metabolism of terpenoids and polyketides	—	*chlP, bchP*
	Phenylalanine metabolism	Amino acid metabolism	*katG*	—
	Spliceosome	Transcription	*HSPA1s; PLRG1, PRL1, PRP46*	—
	Pyruvate metabolism	Carbohydrate metabolism	*E4.1.1.49, pckA; ACSS1_2, acs*	—
	Tryptophan metabolism	Amino acid metabolism	*katG*	—
High concentration vs. Control	Porphyrin and chlorophyll metabolism	Metabolism of cofactors and vitamins	—	*por*
	Biosynthesis of secondary metabolites	Global and overview maps	—	*por*

Supplementary figures

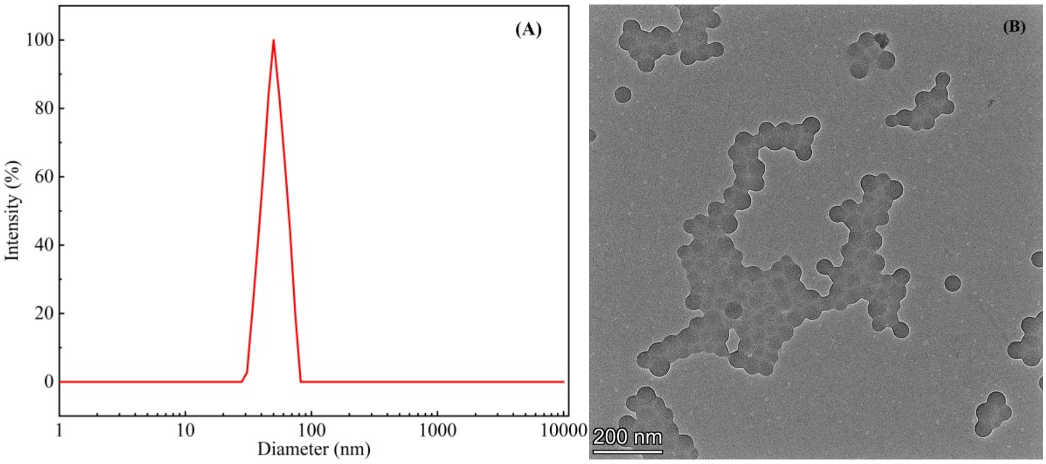

Fig. S1. Characterisations of NH_2-PS and a TEM imaging of PS-NH_2. (A) Particle size distribution characterization of PS-MPs. (B) The transmission electron microscopy (TEM) characterization.

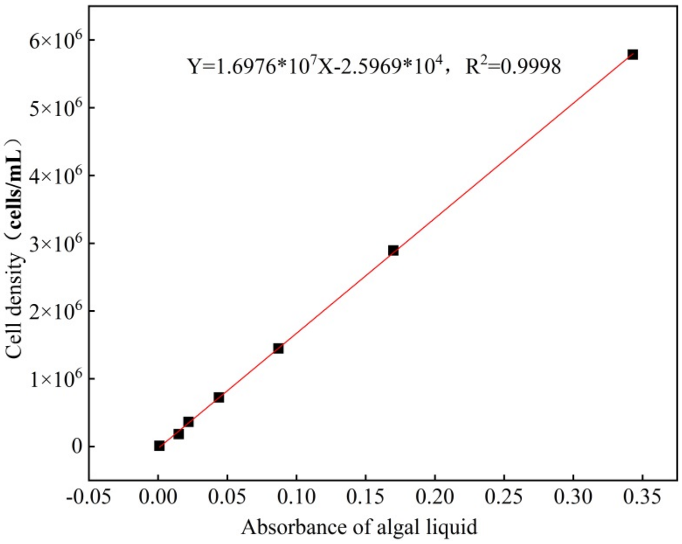

Fig. S2. Standard curve of *Navicula sp*.

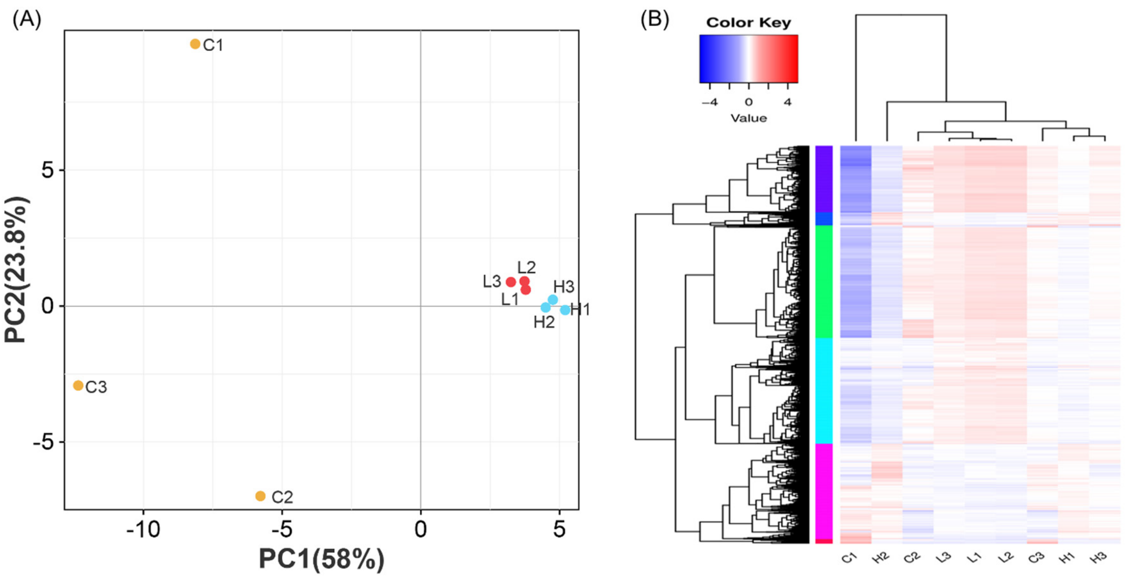

Fig. S3. (A). Characterization of the transcriptome dataset. Principal component analysis (PCA) exhibits clear distinctions between Control and expression group. (B). Volcano map of DEGs in *Navicular sp.* (q-value ≤ 0.05). C: Control group; H: High concentration group; L: Low concentration group.

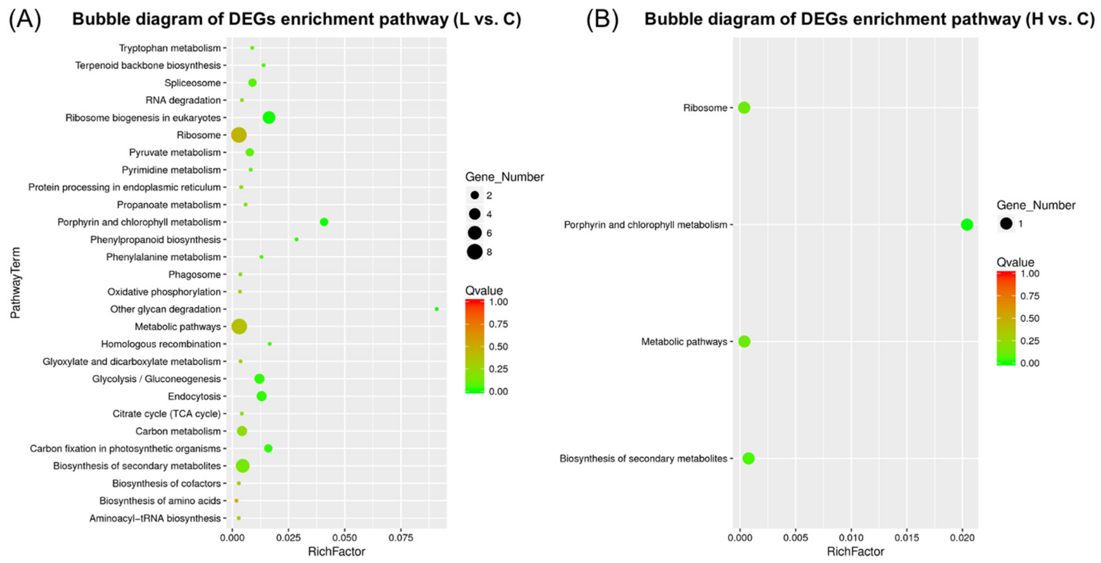

Fig. S4. KEGG pathway changes of *Navicular sp.* induced by PS-NH_2. (A) Low concentration. (B) High concentration. C: Control group; H: High concentration group; L: Low concentration group.

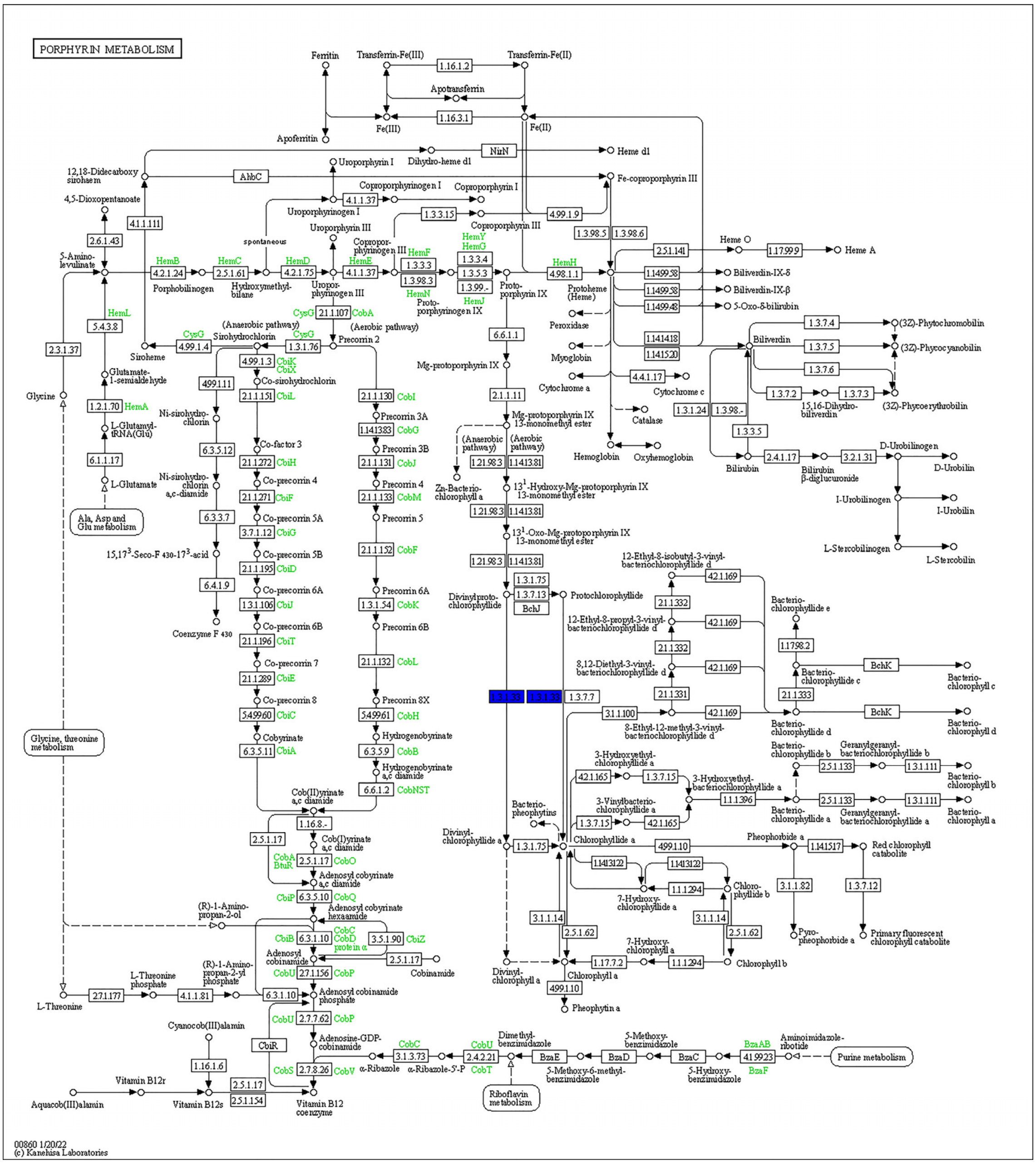

Fig. S5. Transcriptomic analysis of porphyrin and chlorophyll metabolic pathways in *Navicula sp.* after exposure to PS-NH_2.

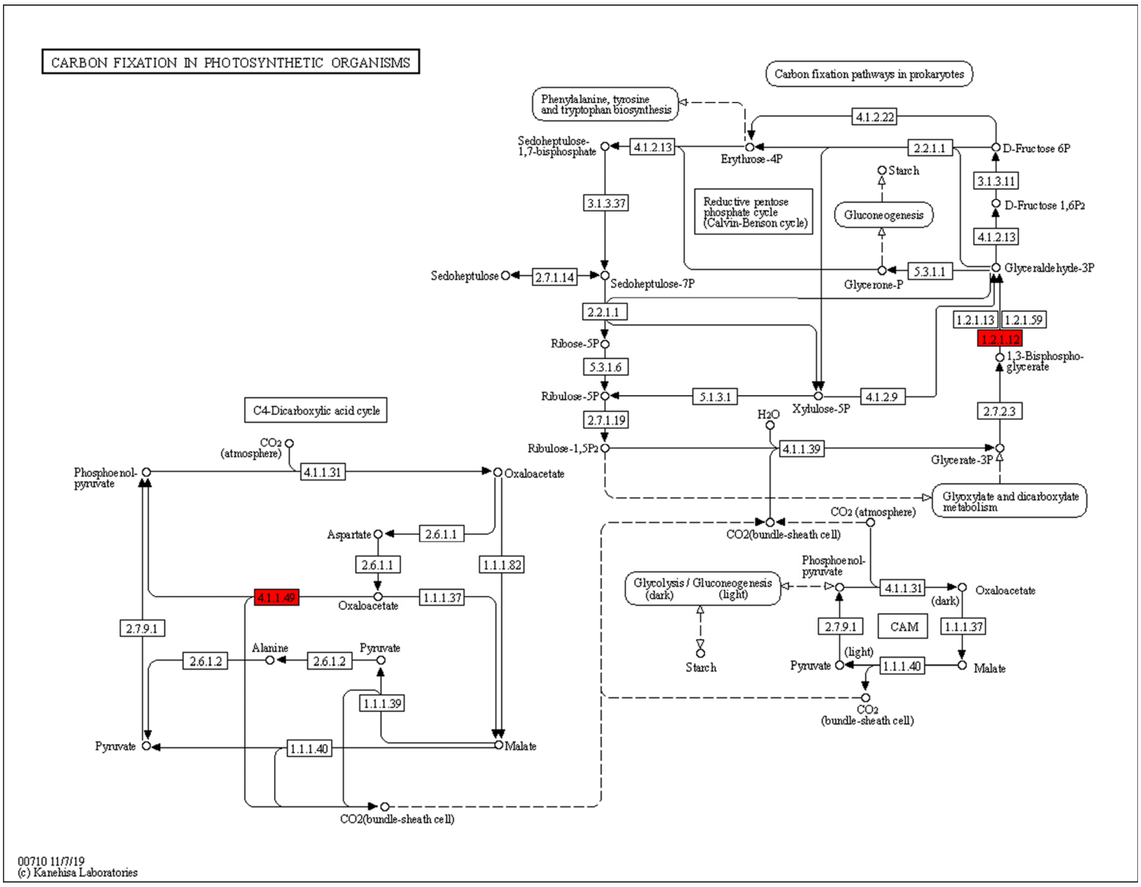

Fig. S6. Transcriptomic analysis of carbon fixation pathways in photosynthetic organisms after exposure of *Navicula sp.* to PS-NH_2.

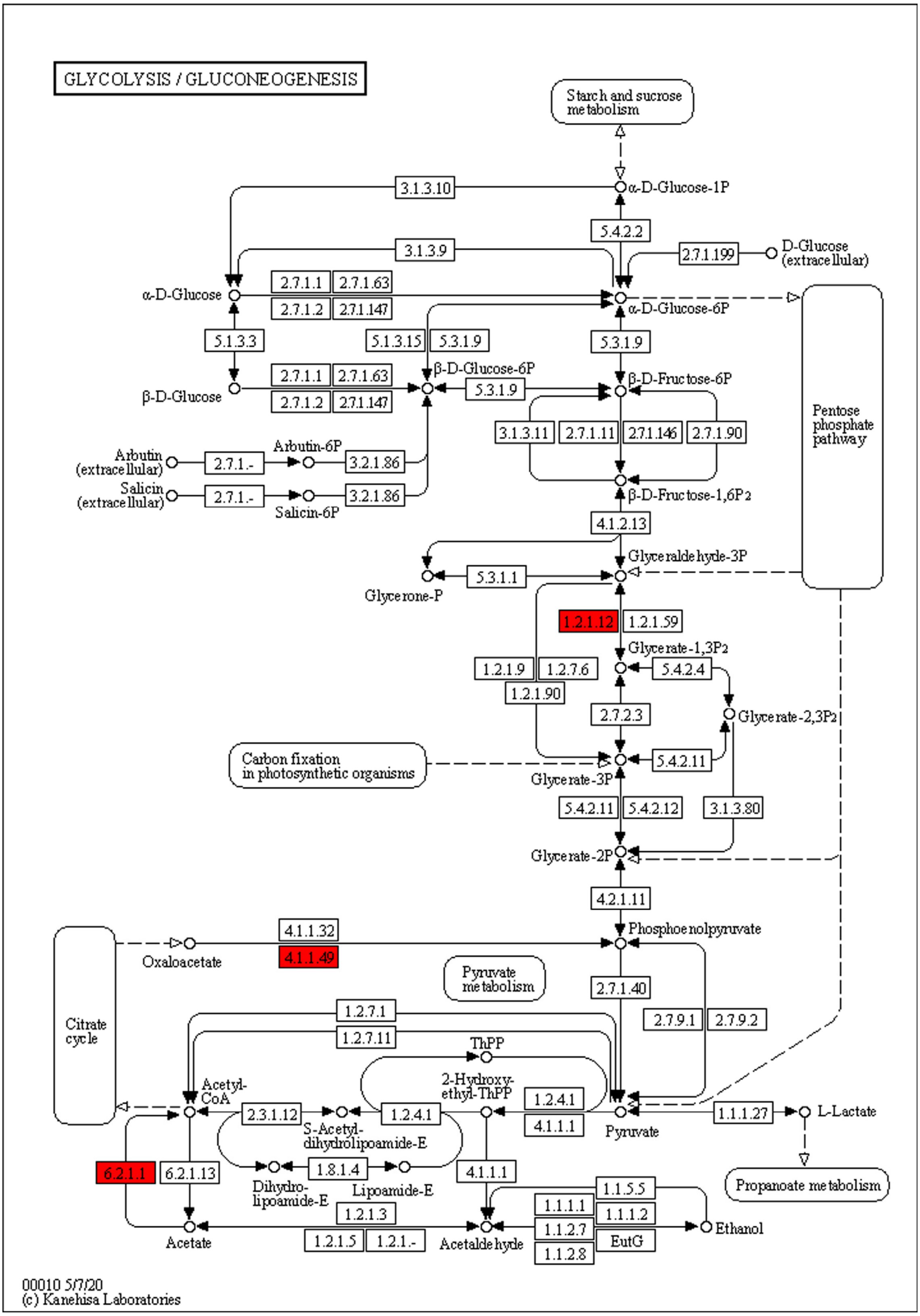

Fig. S7. Transcriptomic analysis of the Glycolytic/Glyco-isomeric pathway after exposure to PS-NH_2 in *Navicula sp.*

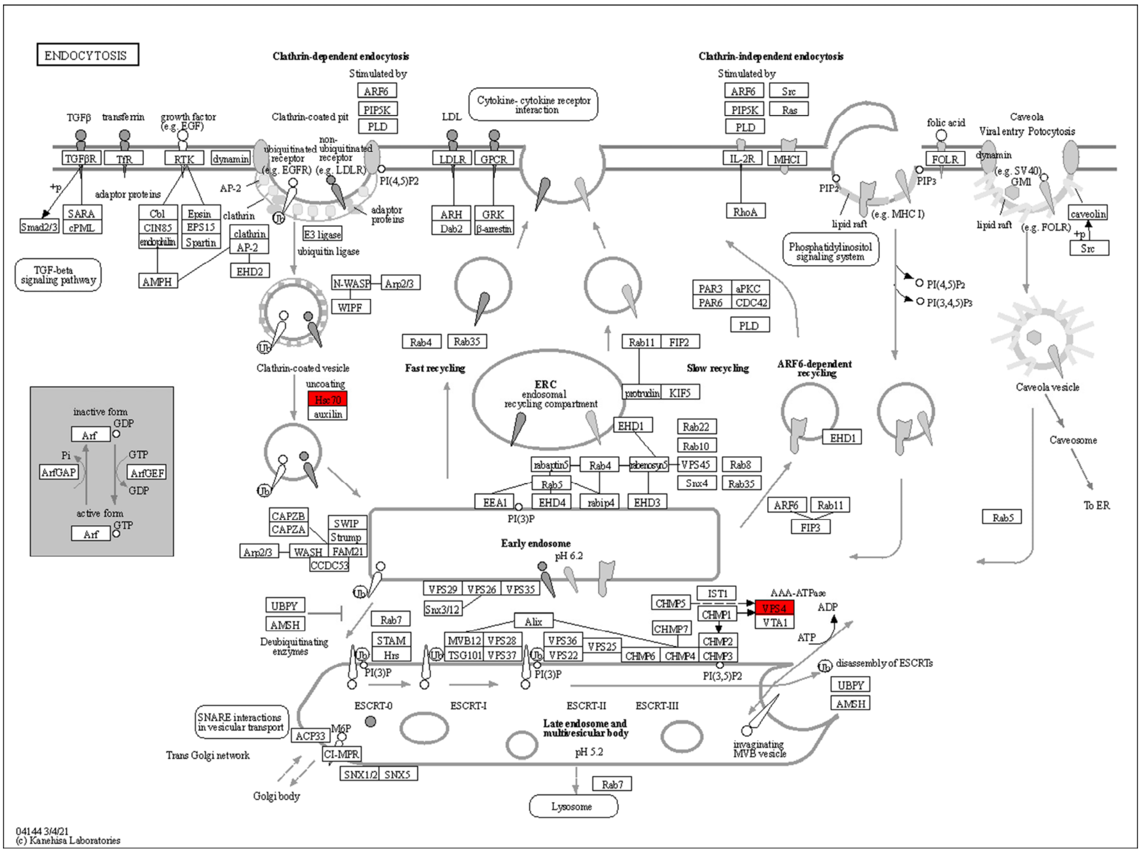

Fig. S8. Transcriptomic analysis of Endocytosis pathway in *Navicula sp.* exposed to PS-NH_2.
